# Waiving the consent requirement to mitigate bias in observational precision medicine research

**DOI:** 10.1186/s12939-024-02221-4

**Published:** 2024-07-18

**Authors:** Ruifeng Song

**Affiliations:** Faculty of Law, The University of Hong Kong, Pokfulam, Hong Kong, China

**Keywords:** Consent bias, Autonomy, Equity, Precision medicine research, Privacy safeguards

## Abstract

Consent bias is a type of selection bias in biomedical research where those consenting to the research differ systematically from those not consenting. It is particularly relevant in precision medicine research because the complexity of these studies prevents certain subgroups from understanding, trusting, and consenting to the research. Because consent bias distorts research findings and causes inequitable distribution of research benefits, scholars propose two types of schemes to reduce consent bias: reforming existing consent models and removing the consent requirement altogether. This study explores the possibility of waiving consent in observational studies using existing data, because they involve fewer risks to participants than clinical trials if privacy safeguards are strengthened. It suggests that data protection mechanisms such as security enhancement and data protection impact assessment should be conducted to protect data privacy of participants in observational studies without consent.

## Background

It is no news that certain population subgroups are structurally left out in biomedical research, including precision medicine research involving genetics and genomics [1]. The exclusion could be attributed to many reasons. Racial or ethnic minorities may have been subject to long-term discrimination in recruitment and enrollment [2]. Individuals from lower socio-economic backgrounds and those less digitally literate may have difficulties accessing technologies that help collect data in research [3]. No matter the reasons, excluding specific subpopulations from research have negative consequences. The direct consequence is that research with unrepresentative samples may lead to potentially biased and invalid research findings. Indirectly, the exclusion prevents an equitable translation of research findings into community health benefits that are as broad as possible. If vulnerable groups are underrepresented in genetic studies, the study recommendations might not fit them. In other words, those unable to participate in the research are also less likely to reap the benefits of precision medicine findings.

### Consent bias

One reason for the exclusion is believed to involve informed consent, the cornerstone of biomedical research ethics [4]. In some studies, research participants that provide consent may be systematically different from those that do not give consent. In these cases, the participants are not representative of the target population, which may lead to biased results. This is known as consent bias [5]. Many factors could account for the consent bias. For instance, while precision medicine research is showing great promise in tackling pediatric oncology with the help of technologies such as high-throughput molecular profiling [6], children may lack the capacity to make decisions and provide consent in the first place [7]. Normally their parents or guardians act on their behalf regarding consent issues. Nevertheless, evidence shows that requiring active parental consent may lead to sampling bias and inaccurate policy recommendations [8, 9]. Another reason for the consent bias might be a lack of trust in medical research or professionals, as is often the case involving ethnic minority participants in geriatric psychiatry research [10]. In these cases, the requirement for consent poses barriers to advancement in medical research.

Despite evidence showing consent bias in biomedical research, Rothstein and Shoben claim that its impact is overestimated and that the bias could be corrected by statistical techniques [11]. This claim is questioned by others because the nature, direction and magnitude of effects due to consent bias are not consistent in different studies, so it is difficult to adjust for biases [12, 13]. The degree of consent bias aside, this debate highlights the controversy over the role of informed consent in biomedical research. Indeed, the call to loosen or even abandon the requirement of consent in at least some categories of research has been voiced by scholars across different disciplines. The issue is often framed as a need to balance the individual interests, including autonomy and privacy, against the public benefits [14–16]. Gostin and Hodge, for instance, advocate a framework that values both privacy and common goods without favoring either *a priori*. They suggest that data may be used for important public purposes (such as reporting infectious diseases to health authorities) without consent, and that individuals do not have the right to veto data sharing for public benefits [14].

Moreover, the debate could proceed in deontological frameworks. On the one hand, there will be arguments that defend inalienable autonomy and underscore the indispensability of consent in whatever research involving human participants [12, 17]. On the other hand, scholars propose ethical frameworks that emphasize a duty to share health data that facilitate research beneficial for others [18, 19]. The debate about “autonomy or community” can go on and on.

Both utilitarian and deontological arguments in these discussions assume that consent is an ideal proxy of autonomy. The problem is that it is not. Real-world consent does not function as it is supposed to. Theoretically, understanding the information disclosed is an element of informed consent [4]. This ideal, however, becomes impractical for three reasons. Firstly, extensive empirical research shows that research participants’ understanding of the disclosure is far from satisfactory [20]. Secondly, because of the rapidly growing need for scientific studies in precision medicine, all potential research needs and purposes may not be anticipated when participants consent [21, 22]. Lastly, the use of emerging technologies may bring risks unforseeable at the time of consent [23]. Therefore, if both future research needs and potential risks are not known when researchers ask for participants’ consent, the understanding level is likely insufficient. Indeed, having “informed” consent in this increasingly complex world becomes more challenging [24]. It is doubtful whether consent should continue to be taken as an appropriate representation of human autonomy. Actually, informed consent is believed to have many more limitations that cast into doubt its centrality in medical ethics [25]. Scholars increasingly ask the question: How to reform the consent model in biomedical research?

### Reforming or removing consent

Existing answers to the above question can be categorized into two groups: reforming consent, or removing it. The reformation camp is further divided into two subgroups. The first aims to improve the means through which research information is communicated so that they become more accessible to participants. And the second focuses on finding alternative consent models that are more practical for consent management.

Improving the quality of communication between researchers and research participants is a strategy to enhance informed consent from parents/guardians in pediatric studies [26, 27]. For instance, presenting information to parents in multimedia forms led to higher comprehension rates of the endoscopic procedures [26]. In the context of precision medicine research, however, the information associated with genetics and epigenetics may be too complex to be presented in straightforward manners. Moreover, because potential data uses and risks are highly uncertain at the time of consent, it is simply impossible to communicate those unknown possibilities.

A range of alternative consent models has been proposed to replace the traditional model, including dynamic consent, meta-consent, opt-out consent, broad consent, and consent for governance [28–32]. The comparative advantages and disadvantages of these models are hotly debated, and admittedly none of them cures the problems of consent once and for all. More importantly, each of these models potentially creates equity problems. Dynamic consent requires constant communication about new uses of data to research participants, and that would impose additional costs on those who are digitally illiterate or lack access to communication tools. In opt-out consent schemes, inaction signals consent, and participants are offered the option to quit anytime. While evidence shows lower consent bias with this scheme [33], some versions of the model become very similar to the opt-in model because sufficient information about the research and opt-out procedures must be communicated to participants for them to make informed opt-out decisions. In broad consent scenarios, trust becomes essential for research participants who are asked to agree to all future uses of health data. But if the trust deficit is already significant in specific consent contexts, why would participants agree to a scheme with higher uncertainty? Lastly, these potential problems may be magnified in the data-intensive precision medicine research such as the All of Us Program in the US where the number of participants to be recruited will be many times that of a regular clinical trial, and where the nature of data involved is highly sensitive. Therefore, researchers should ensure that the use of these alternative consent models do not further amplify consent bias. For instance, the decision to use dynamic consent should be supported by evidence on the willingness and capacity of different population subgroups to engage with digital tools [34]. More empirical research is needed to provide such evidence.

Another problem of various consent models is that they create burdens on both sides of the research. On the one hand, consent places the burden of understanding the research and risks on the part of the participants. It is a task inappropriate for them because of the expertise normally required to fully comprehend biomedical research. On the other hand, the requirement for consent imposes significant administration costs on researchers. These costs are not negligible in cohort studies on precision medicine that aim to include over 1 million participants [35]. It does not seem to be a win-win situation.

Given the deficiencies of consent, there have been calls to remove the requirement of consent, at least in certain categories of research [14, 15, 36]. As noted above, the main argument is that we should strike a balance between upholding individual autonomy and advancing public health benefits through research. The argument is especially appealing in observational studies where the risks are considered lower than in interventional research.

### Waiving consent in observational studies using existing data

Although randomized clinical trials are regarded as the “gold standard” for evidence-based research [37], they are not always possible or desirable. Observational studies based on existing databases provide unique value to precision medicine research when clinical evidence is limited [38, 39]. But the validity of observational studies may also be affected by consent bias [40]. Considering solutions to consent bias in observational studies is an easier task than tackling bias in clinical trials because fewer interests of participants are affected in observational studies which by nature are non-interventional.

In terms of potential harms on research participants, these two types of research differ significantly. Because observational studies do not involve direct intervention for participants, they usually do not affect interests such as health or bodily integrity of participants. When observational studies use existing health records instead of primary data, they do not even involve interaction with the participants. The potential harms on participants are principally related to their autonomy and privacy. Indeed, informed consent was originally established as a guardrail mainly to prevent abusive experiments involving human beings, not purely observational research using existing data [36, 41].

The autonomy of participants is relevant in observational studies using existing data because the participants are supposed to have the capacity to choose whether they provide their data for the research. Where they have provided broad consent to future research when their electronic health records were created, it may not be necessary to obtain their separate consent for a new observational study. Without broad consent, however, a separate consent to access existing data is necessary under most circumstances. Fulfilling this requirement may be onerous, costly, and conducive to selection bias. Is it desirable for autonomous participants to be able to say no to any study even if it merely uses their existing records without interference into their lives?

Privacy of participants is another major factor to be considered. Health data is highly sensitive. The abuse of these data may cause dignitary, reputational, psychological or even financial harms to participants [41], who certainly have the right to defend themselves against these risks. But it is important to note that these harms are derivative of the erosion of data privacy. If privacy is guarded well and these derivative harms are unlikely to occur, is it desirable to consider waiving the consent requirement?

Answering the above questions requires weighing the costs of obtaining consent against the benefits of waiving it. On the one hand, waiving consent helps mitigate the selection bias and save costs. On the other hand, it may undermine the autonomy or privacy of participants. But there are more factors to be taken into account. The loss of individual autonomy to decide whether to participate in a study should be balanced against the societal gains, which are likely higher when the research focuses on public health emergencies such as the COVID-19 pandemic. Furthermore, the threat to individual privacy may be mitigated if technical and organizational security measures function adequately so that the risk of privacy infringement and other derivative harms remains low. In observational studies, compromises made on autonomy and privacy issues are more likely to be compensated by the gains, on condition that suitable safeguards are in place.

### The safeguards for data privacy

If compromises can be made about participants’ autonomy (i.e. their capacity to refuse to consent) in observational studies, the remaining key issue is for researchers to take good care of participants’ data privacy. This means the implementation of a series of technical and organizational measures. The General Data Protection Regulation (GDPR) of the European Union [42], the most influential data protection law in the world, provides a series of such mechanisms, such as security of data processing (Article 32), data breach notification (Articles 33 and 34), and the data protection impact assessment (Article 35). Security of data processing requires that researchers adopt measures to ensure a level of security proportionate to the risk. These measures include data encryption and the maintenance of a resilient data processing system. Data breach notification means that if a data breach occurs and is likely to result in a high risk, researchers should notify both the data protection authority and participants. Data protection impact assessment (DPIA) is one of the most important mechanisms in the law. It mandates that large-scale processing of sensitive data must be conducted after a DPIA, which evaluates the risks and impact on privacy of the data processing activity. Because health data is considered sensitive data, it is likely that researchers processing health data on a large-scale would need to conduct a DPIA. If the DPIA shows a high risk and the researchers’ security measures are insufficient, they should consult the data protection authority and seek guidance (Article 36). What these mechanisms have in common is that they place the burden of data protection on the part of researchers, not participants. Even though the GDPR is already far-reaching, it is necessary to reiterate the desirability of these mechanisms in guarding data integrity.

These mechanisms operate independent of consent. If we liken consent to a guard of entrance that tries to stop anything illegitimate from entering the room, these mechanisms are the guards within the room that prevent illegitimate things (unethical or illegal studies) from messing around in the room. The guards in the room play their roles regardless of whether the entrance guard successfully screens the entrants.

These safeguards do not eliminate the need to obtain consent in all observational precision medicine research. For observational studies that use primary data collected by researchers, consent remains necessary to invite collaboration of potential participants in the first place. Even for studies based on secondary use of existing health records, which this Comment mainly addresses, evaluations should be made to compare the risks to participants and the benefits of the research. These evaluations may be conducted in the form of a DPIA as required by the GDPR or other national laws, or in accordance with formalities required by Institutional Review Board (IRB) policies and procedures. What is important is that research ethics policies start to recognize the possibility or even desirability to waive the consent requirement in at least some of the observational studies.

## Conclusions

Admittedly, there is no one-size-fits-all for tackling consent bias and enhancing equity in precision medicine research. This Comment argues that because fewer interests of participants are at stake in observational studies using existing data, waiving the consent requirement may be a desirable option to mitigate consent bias in these studies. But this must be accompanied by strictly implemented technical and organizational data protection measures lest the data privacy of participants is compromised.

Future studies may take multiple paths. First, they can provide empirical evidence on the desirability of waiving consent requirements in certain studies. Ideally, randomized trials can be conducted to compare the outcomes of different consent schemes [43]. Second, they can study the mechanisms for researchers to give an account of their actual data processing activities and data protection measures. These accounts should help researchers build trust with research participants who may wish to know whether their data are being taken care of. Lastly, legal scholars should continue to explore how to apply sanctions in data protection laws to research settings when researchers fail to implement the safeguards, especially who bears liabilities if privacy harms occur.

## Data Availability

Not applicable.
